# Immunophenotypic characterisation of morphologically diagnosed cases of Acute Myeloid Leukaemia (AML)

**DOI:** 10.12669/pjms.35.2.614

**Published:** 2019

**Authors:** Maria Basharat, Saleem Ahmed Khan, Nasir ud din, Dawood Ahmed

**Affiliations:** 1 *Maria Basharat, MBBS. Pathology Department, Army Medical College, National University of Medical Sciences, Islamabad, Pakistan*; 2 *Saleem Ahmed Khan, MBBS, MCPS, FCPS, FRCP, PhD. Pathology Department, Army Medical College, National University of Medical Sciences, Islamabad, Pakistan*; 3 *Nasir ud din MBBS, FCPS. Pathology Department, Army Medical College, National University of Medical Sciences, Islamabad, Pakistan*; 4 *Dawood Ahmed MBBS, FCPS. Pathology Department, Army Medical College, National University of Medical Sciences, Islamabad, Pakistan*

**Keywords:** Immunophenotyping, Acute myeloid leukemia (AML), Aberrant phenotype in AML, Flowcytometry

## Abstract

**Objective:**

To determine immunophenotypic pattern in newly diagnosed cases of acute myeloid leukaemia by flow cytometry and its correlation with morphological findings.

**Methods:**

This study was conducted at Haematology (Pathology) department, Army Medical College, in collaboration with Immunology Department Armed Forces Institute of Pathology, Rawalpindi from 16 November 2016 to 16 November 2017. One hundred and six patients of both genders and all age groups diagnosed as acute myeloid leukaemia were included in the study. Demographic data was noted. Complete blood counts, bone marrow examination and cytochemical stains were carried out and evaluated microscopically for blast percentage and morphology. Immunophenotyping was performed by flow cytometry using standard panel on peripheral blood or bone marrow samples. The surface and cytoplasmic antigens of interest were analysed and correlated with morphological findings.

**Results:**

The most commonly expressed antigens were CD13, CD33, CD45 and HLA-DR. Almost all blasts expressed CD45 with no remarkable difference among the subtypes of AML. The mean positivity for CD13 among all AML subtypes was 57% and for CD33 was 67%. Aberrant expression of CD7 and CD19 were expressed in 26.4% and 1.1% of all cases respectively. There was concordance rate of 90% between morphology and FCM in our study.

**Conclusion:**

Flow cytometric analysis of acute leukaemia done by a combination of patterns and intensity of antigen expression improves diagnostic yield in AML. CD13, CD33 and CD45 are the most frequently expressed antigens in AML. Our findings suggest a 90% concordance between morphology and flow cytometry. It is pertinent to conclude that flow cytometry results interpreted with morphology are complementary.

## INTRODUCTION

Acute Myeloid Leukemia (AML) is a clonal disorder of haemopoietic stem cells. It is characterised by inhibition of differentiation resulting in accumulation of cells at various stages of incomplete maturation. There is a decreased production of mature haemopoietic elements.^1^ This relatively common haematological malignancy comprises 80% of acute leukaemias in adults and 20% in children.^2^ Being the second most common type of leukaemia in the United States, studies have shown AML to be the commonest leukaemia in Pakistan.^3^

Diagnostic methods for acute leukemias include immature cell count, morphology, cytochemistry, immunophenotyping, cytogenetics and histochemistry in correlation with clinical features. All these diagnostic methods are complementary. Cell morphology remains the basic diagnostic tool to assess the number and morphology of blasts. Immunophenotyping by flow cytometry is a powerful adjuvant tool in delineating cell surface and cytoplasmic markers in AML. The expression of characteristic myeloid lineage markers CD13, CD33 and CD117 allows the distinction of AML from other types of leukaemias.^4^

In the diagnosis of acute leukemia concordance between experienced observers increases from 70 to 99% when morphologic criteria are supplemented by cytochemical and immunophenotypic information.^5^ Immunophenotyping plays an important role when morphological interpretation is difficult. The main advantage of immunophenotyping is identification of particular leukaemia subtype that cannot be identified by morphological criteria alone. Immunophenotyping of peripheral blood and bone marrow in leukaemia determines the decision making for a specific therapeutic regimen and is a practical prognostic indicator.^6^ The diagnosis and management of acute leukaemia depends primarily on the detection, identification and characterization of leukemic cells.^7^

Immunophenotypic markers shown in various studies implicating adverse outcomes are CD7, CD9, CD11b, CD13, CD14, CD33, CD34, CD56 and terminal deoxynucleotidyl transferase.^8^ Regardless of the increasing importance of molecular and genetic features in the sub classification of acute leukemias, morphology and immunophenotyping remain the primary modalities by which leukemias are evaluated.^9^

Aberrant phenotype is expression of lymphoid-associated markers in myeloblasts or that of myeloid associated markers in lymphoblasts.^7^ Aberrant immunophenotypic expression has been used to predict treatment outcome.^10^ The aim of our study was to determine immunophenotypic patterns of de novo AML cases by flow cytometry and to correlate with morphological, French American British (FAB) classification. Rationale of this study was to characterize immunophenotypic pattern of newly diagnosed patients of AML by flow cytometry (FCM) so that the diagnosis of AML can be improved and its correlation with morphological findings.

## METHODS

This descriptive cross sectional study was carried out in Haematology Department Army Medical College in collaboration with Haematology and Immunology department of Armed forces institute of pathology, Rawalpindi from 16 November 2016 to 16 November 2017. The sampling technique used was non probability convenient sampling Approval was taken from ethical review board and institutional review board. A total of 106 patients of all ages and both genders who were newly diagnosed with AML were included. Relapsed cases of AML and those evolving from myelodysplasia (MDS) or receiving treatment for AML were excluded.

The samples were analysed for blast percentage and morphology on leishman stained peripheral blood and bone marrow smears followed by cytochemicalstains (Sudan Black B). Immunophenotyping was done using three colour flow cytometer. This included samples of peripheral blood or bone marrow aspirate from patients of De novo AML.

### Sample Collection and Preparation

Two and a half ml of venous blood in Ethylene Diamine Tetra Acetic Acid (EDTA) tube was collected for Complete Blood Count (CBC) and peripheral blood film examination, under aseptic conditions. Bone marrow aspiration was done after written informed consent, following standard guidelines. Complete blood counts were generated using automated haematology analyzer Sysmex KX-21. Leishman stained smears from peripheral blood and bone marrow aspirates were examined under microscope for morphology and percentage of blasts.^11^

### Lysing of Whole Blood and Combination of B.M Aspiration, Monoclonal Antibody

Labelling of each test tube (Falcon, BD) was done properly and placed in sequence. Ten µ| of monoclonal antibody was added into each tube. 50µl of whole blood / diluted bone marrow in each tube was added, thoroughly mixed and incubated in dark for 30 minutes at room temperature. Fluorescent activated cell sorter lysing solution (FACSLyse) in distilled water was prepared in 1:10 dilution. In each test tube 2 ml of diluted FACSLyse was added and incubated in dark for five minutes at room temperature. It was centrifuged at 300 g for five minutes at room temperature. The supernatant was discarded and the remaining 50µ| fluid was shaken for resuspension of cells. In each tube 2ml of phosphate buffered saline (PBS) was added. Centrifuge at 300 g for five minutes and supernatant was discarded. The remaining fluid was shaken and 0.5 ml of 3.3% formalin was added to each test tube. The samples were kept at 4ºC till analysis on flow cytometer.

### Immunophenotyping by flow cytometry

Flow cytometric analysis was made by a top FACScan flow cytometer (Becton & Dickenson).The flourochromes FITC, PE and PerCP were used. The primary panel of monoclonal antibodies (mAbs) used were CD45 (for blast identification), CD13, CD33, CD34, HLA-DR and for AML M4/M5 CD14, for AML M6 anti-glycophorin A and for AML-M7, CD61, CD41 and CD42 were used. The extended panel included CD117 and Anti-MPO was used in cases where the morphology suggested AML-M_0_ and where results of primary panel were inconclusive. The populations were considered positive if at least 20% of cells within gate showed the expression of a particular antigen. For cytoplasmic antigen expression, the threshold was 10%.

### Quality control

Isotype control (antimouse IgG1FITC/Ig2αPE) is used as negative control (should not react with human blood/bone marrow cells). If it happens, the test/reagent should be rechecked.

## RESULTS

Data was analyzed using SPSS version 21. Frequencies and percentages were calculated for variables like gender. For quantitative data analysis, mean along with standard deviation were used to assess the values for monoclonal antibodies Correlation tests were applied using Pearson correlation coefficient. The age of the patients at the time of diagnosis ranged from one year to 80 years (mean age = 31.81±19.972 years). Males constituted 58.5% and females were 41.50%. The male to female ratio was 1.4:1.

The mean haemoglobin levels were 8.25±2.16 g/dl. The lowest haemoglobin level was 3g/dl seen in AML-M2 and highest levels in AML-M3 of 13.50 g/dl. The mean TLC was 57.46±79.39, the lowest being 0.99 x 10^9^ /L and highest being 456.77x10^9^/L both extremes seen in AML-M1.The mean of platelet count was 58.23±81.29 × 10^9^ /L. The highest count was 1000 ×10^9^ /L seen in AML-M2 and lowest being 659 × 10^9^ seen in AML-M1.

**Fig.1:**
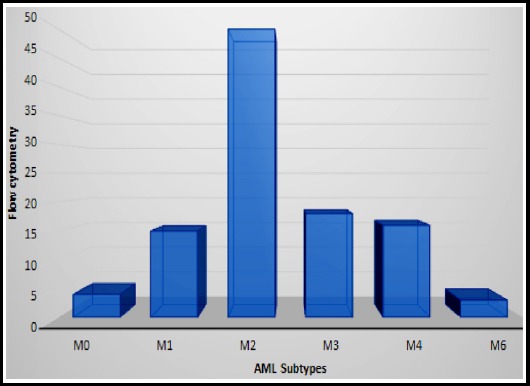
Frequency of subtypes of AML by immunophenotyping.

The least number of blasts were 22(22%) seen in AML-M2 and AML-M6 and highest abnormal promyelocytes with reniform nuclei were 98 %, found in AML-M3. Out of 106 cases analyzed, AML-M2 was the most frequent subtype constituting 49 (47.2%) cases followed by AML-M3 (n=18, 17%,). The least common being AML-M6 (n=3, 2.8%) as shown in Fig.1. All cases expressed CD45 with mean positivity of 70.42% ± 23.74 and no significant variations among the subtypes as shown in Table-I. HLA-DR was expressed in 51.49% ± 33.92 of all AML cases. The strongest positivity was seen in AML-M_O_ (75%±33.42) and weakest positivity in AML-M3 (12%±23.84). CD34 showed mean positivity of 38% ± 30.84 among all AML subtypes, strongest being in AML-M_O_ (74% ± 21.79) and weakest seen in AML-M6 (1.33% ± 2.31). In our study there were 18 cases (n =106) of AML-M3 of which only 1 case of AML-M3 showed positivity for HLA-DR and 3 cases (n=18) were positive for CD34, however none of them were positive for both CD34 and HLA-DR. CD13 was studied among the blast population with mean positivity of 57% ±27.53 among all subtypes with strongest positivity in AML-M4 (72% ± 20.01) and weakest in AML-M6 (22% ± 1.73).

**Table-I:** Distribution of positive results of different markers among different AML subtypes.

AML Subtype (FCM)	N	Weak CD45	HLA-DR	CD 13	CD 33	CD 34	CD 14	CD7
AML M0	4	82.0%	75%	64.00%	49.75%	73.75%	0.0%	0.0%
AML M1	15	79%	70%	56.00%	72.53%	45.33%	0.0%	27.06%
AML M2	49	71.24%	57%	50.53%	60.18%	44.81%	0.0%	13%
AML M3	19	82%	12%	64.73%	72.52%	11.68%	0.0%	1.48%
AML M4	16	52%	62.8%	71.812	86.81%	38.06%	44.12%	0.00
AML M6	3	29.33%	28%	22.00%	30.00%	1.33%	0.0%	15.0%

Total	106	70.42%	51.49%	56.71%	66.91%	37.7925	6.72%	10.33%

**Fig.2:**
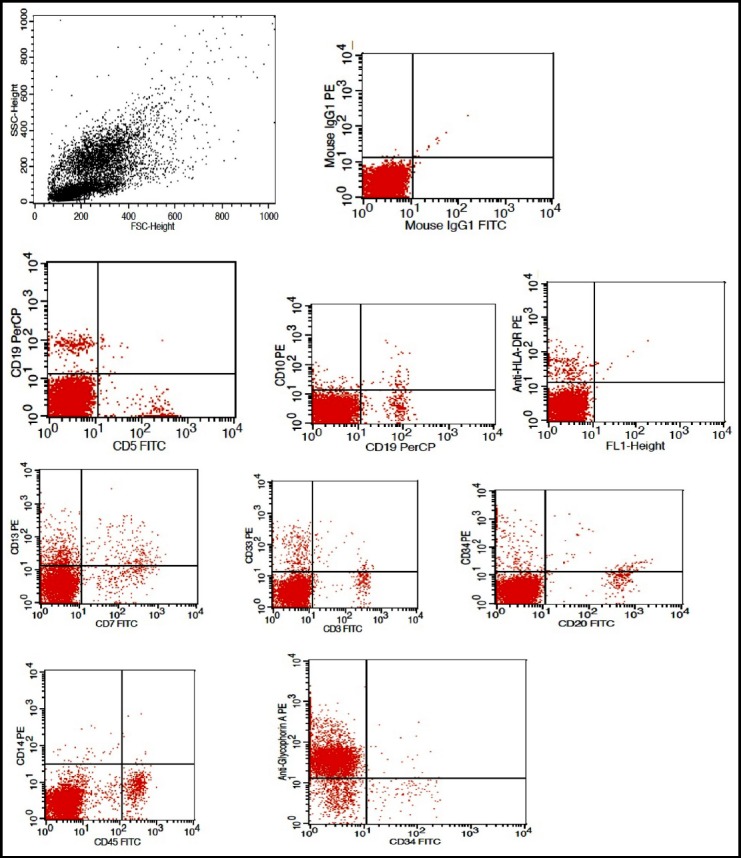
Flow cytometric findings in a case of AML-M6.

CD33 showed mean positivity of 67% ± 27.56, strongest being in AML-M4 (87% ±9.02) weakest in AML-M6 (30% ±11.14).CD13 and CD33 were also studied for correlation using Pearson correlation and was found to be statistically significant among all subtypes. The strongest correlation being in AML-M6 (r= 0.962) and weakest being in AML-M_0_ (r=0.717).

The concordance rate between morphology and FCM is seen in 102 (90%) cases with confidence interval of 95% in our study. While partial discordance was seen in 4(3.77%) cases where lineage was correctly identified with difference in defining subtypes only.

The aberrant expression of CD7 showed positivity in 28 (26.4%) cases, most frequently expressed in AML-M2 and CD19 was expressed in 1(1.1%) case. The results of correlational analysis showed that CD 13, CD 34, CD7 and CD14 had a significant correlation with immunophenotyping (*r =-.321, .360 .297 and -.585* respectively) and insignificant correlation with CD33, CD117 and CD19 (r=.111, .141 and .031 respectively).

## DISCUSSION

Immunophenotyping has a recognised role in the diagnosis and classification of acute leukaemia.^12^ AML has an age adjusted incidence of 3.7/100,000 per annum in US with highest incidence in 7^th^ decade.^3^ AML can occur at any age group but the incidence increases with age.^2^ The mean age in our study was 32 years similar to Harani et al.^5^; However, in some other studies mean age was more than our study ranging from 35 to 47 years.(2,3,13-15)

Childhood AML comprised 27.3% in our study while adult AML comprised 72% which was slightly different from Gosh study. (24%,76% respectively).^16^ Conversely, in Ahmad et al., paediatric cases were 20% and adults 86.4%,^3^ There is a predilection for men with AML, 4.8 versus 3.3 new cases whereas in ALL, there is no gender variance (1.9 new cases in men and 1.5 in women).^17^ In our study the male to female ratio was 1.2:1 indicating a slight male predominance is similar to some other international studies.^2^,^5^,^13^,^16^,^18^ In Patel et al., the male to female ratio was 1:1.1.^19^

In our study AML-M2 (47.2%) was the most frequent subtype similar to Gosh study (AML-M2=34%).^16^ Conversely, in few other international studies^3^,^5^,^14^, other AML subtypes predominated.

CD7, a T-cell antigen known to show aberrant expression was most commonly expressed in our study (26.4%) followed by CD19 (1.2%). The same trend was observed in most of the international studies where CD7 was most commonly expressed aberrant antigen followed by CD19.^2^,^8^,^14^,^16^,^20^ CD7 expression in AML is correlated with lower incidence of complete remission.^2^ According to Belurkar et al., expression of lymphoid associated antigens except CD7, on AML blasts lack prognostic significance and CD7 + AML is a particular subset but in general, it may not represent a biologically distinct form of leukemia since these cases have similar clinical features and a comparable response to therapy.^21^

The combined use of CD34 and HLA-DR was more helpful in distinguishing Acute Promyelocytic Leukemia (APL) from non-Acute Promyelocytic Leukemia (non-APL) none of the eighteen cases of APL were positive for both CD34 and HLA-DR whereas 64.77% of non-APL (83%) were positive for both CD34 and HLA-DR. While out of eighty-eight non-APL, two (2.27%) cases were negative for both HLA-DR and CD34 as compared to 77.87% of APL. Thus the negativity for these two antigens doesn’t refer to APL diagnosis exclusively.

For more than 20 years, the FAB classification for acute leukemia has been the major system of classification. This classification enabled the diagnosis of a variety of morphologic and cytochemical subtypes of acute leukemia through a structured criterion. However, studies are indicative of failure of most of categories of FAB system in delineating significant disease groups based on morphology and cytochemistry in terms of survival of patients.^21^ In our study we analyzed 106 cases of AML by FCM and compared them with their morphological (FAB) diagnosis as well as frequency of their immunological patterns. There was concordance rate of 90% between morphology and FCM of AML while partial discordance rate was 3.77%.

In case 1, morphological diagnosis was that of AML-M3 while on FCM; HLA-DR and CD34 were positive and was reported as AML-M2. In case 2, morphology suggested AML-M5 but FCM showed negative expression for HLA-DR, CD34, and CD14, reported as AML-M3. Morphological diagnosis of case 3 was AML-M6 but FCM showed negativity for anti-glycophorin A and was reported as AML-M2. Morphology in case 4 was suggestive of AML-M2. However, on FCM positivity of CD14 suggested AML-M4. This discordance was resolved on cytogenetic and molecular analysis. Morphological analysis along with cytochemical stain (SBB) rendered the diagnosis in >80% of our AML cases. Similar study was carried out by Berlukar et al.^21^, where the complete concordance rate was 58%, partial concordance 22%. In Kheiri et al., the concordance rate was 77.4% with 89.2% of the myeloid leukemias showing agreement between morphology and FCM^22^, in Mhawech et al., the concordance rate was 80.32%.^23^ The difference between our study and the above mentioned studies is that we included only AML while these studies compared the morphology and FCM of acute leukemias that is both ALL and AML which explains high concordance rate in our study. Morphology and immunophenotyping complement each other mainly because they have as common objects malignant cell phenotype as a whole (morphology, i.e. surface and intracellular marker expression). Conversely, morphology is burdened by a high degree of subjectivity and flow cytometry techniques have not consensus standard protocols yet. That is why correlation of results provided by the two techniques is still absolutely necessary.^24^

### Limitations of the study

One of the limitations was that this study was conducted on a relatively small sample size. the specific markers for AML were used but there was limited use of markers like MPO and CD117. Both these markers are one of the most sensitive markers of myeloid lineage. These were used in only a few cases. Moreover this study would improve if parallel use of molecular analysis was done. However, there were only very few cases with molecular analysis.

## CONCLUSION

It is concluded that immunophenotyping is an essential tool in diagnosis and classification of AML. Our findings suggest a 90% concordance between morphology and flow cytometry However, it depends upon the availability of facility for broad panel of primary and secondary markers, which is available only in specialized centres. Interpretation of immunophenotyping should be done in close correlation with morphological findings as both these complement each other. It is need of the hour to extend facility of immunophenotyping at regional levels in the country so that access to these sophisticated facilities is available to patients of leukemias to avoid long distance travel by members of this diseased community.

## RECOMMENDATIONS

Our findings suggest complementary use of immunophenotyping with morphology improves outcome in the diagnosis of acute leukemias. Further research with use of extended panel of monoclonal antibodies is recommended. Additionally, development of complementary diagnostic techniques such as cytogenetics and molecular analysis should be developed for more precise diagnosis of AML.

### Authors’ Contribution

**MB:** Did data collection, statistical analysis & and manuscript writing.

**SAK:** Conceived, designed, review and final approval of manuscript.

**ND:** Did statistical analysis, review & editing of manuscript.

**DA:** Did statistical analysis & review of manuscript.
